# A Polyoxometalate-Encapsulated Metal–Organic Framework Nanoplatform for Synergistic Photothermal–Chemotherapy and Anti-Inflammation of Ovarian Cancer

**DOI:** 10.3390/molecules27238350

**Published:** 2022-11-30

**Authors:** Diqing Wang, Yuqi Wang, Xinyu Zhang, Qian Lv, Guiqi Ma, Yuan Gao, Shuangqing Liu, Chenyu Wang, Changzhong Li, Xiao Sun, Jipeng Wan

**Affiliations:** 1 Department of Gynecology, Shandong Provincial Hospital Affiliated to Shandong First Medical University, Jinan 250021, China; 2 School of Chemistry and Pharmaceutical Engineering, Medical Science and Technology Innovation Center, Shandong First Medical University and Shandong Academy of Medical Sciences, Jinan 250000, China; 3 Department of Obstetrics and Gynecology, Peking University Shenzhen Hospital, Shenzhen 518036, China

**Keywords:** polyoxometalate, PTT, anti-autophagy, anti-inflammatory, cancer therapy

## Abstract

Photothermal therapy (PTT), as a noninvasive and local treatment, has emerged as a promising anti-tumor strategy with minimal damage to normal tissue under spatiotemporally controllable irradiation. However, the necrosis of cancer cells during PTT will induce an inflammatory reaction, which may motivate tumor regeneration and resistance to therapy. In this study, polyoxometalates and a chloroquine diphosphate (CQ) co-loaded metal–organic framework nanoplatform with hyaluronic acid coating was constructed for efficient ovarian cancer therapy and anti-inflammation. Our results demonstrated that this nanoplatform not only displayed considerable photothermal therapeutic capacity under 808 nm near-infrared laser, but also had an impressive anti-inflammatory capacity by scavenging reactive oxygen species in the tumor microenvironment. CQ with pH dependence was used for the deacidification of lysosomes and the inhibition of autophagy, cutting off a self-protection pathway induced by cell necrosis–autophagy, and achieving the synergistic treatment of tumors. Therefore, we combined the excellent properties of these materials to synthesize a nanoplatform and explored its therapeutic effects in various aspects. This work provides a promising novel prospect for PTT/anti-inflammation/anti-autophagy combinations for efficient ovarian cancer treatment through the fine tuning of material design.

## 1. Introduction

Ovarian cancer is currently the number one cause of death associated with gynecologic malignancies worldwide [1]. Common clinical treatment modalities include surgery and drug therapy. Due to the high malignancy of ovarian cancer, the survival rate of patients with advanced disease remains low, even with operation and conventional chemotherapy [2]. This remarkably high mortality rate is due to many reasons, which include postoperative metastasis or tumor recurrence, polypharmacy resistance, and nontargeted drug delivery [2,3]. Therefore, there is a necessity to explore new therapeutic strategies to meet the challenges of ovarian cancer treatment. Numerous nanomaterials such as two-dimensional nanomaterials and precious metal nanomaterials have been found to possess the feature of causing an increase in temperature when exposed to light, which is capable of killing tumor cells [4]. This effect leads to photothermal therapy (PTT), which has become an effective substitute or supplementation tool in oncology treatment [5,6,7]. As a non-invasive local treatment option, PTT can be an effective way to abolish tumor tissues without causing any whole-body toxicity or tissue damage [8]. Though PTT has achieved immediate tumor ablation, the release of intracellular components can lead to adverse inflammatory reactions, which hinders the therapeutic effects of PTT [9,10,11]. Although the inflammatory process is generally considered to be a self-limiting tissue repair response, the dysregulation of chronic inflammation may also inhibit the formation of anti-tumor immune responses and the microenvironment of tumor proliferation, which becomes one of the reasons for tumor occurrence and development [9]. At the same time, concomitant tissue damage from cancer therapy, cellular necrosis, and even infection by external pathogens can trigger secondary inflammatory responses, which may also to a certain extent stimulate tumor regeneration and impact the effectiveness of tumor treatment [12]. It has been confirmed that the accumulation of highly reactive oxygen species (ROS) such as hydrogen peroxide (H_2_O_2_) plays a significant role in stimulating tumor recurrence and destroying peripheral normal cells [13]. Consequently, a PTT nanoagent with an anti-inflammatory function would be ideal in the treatment of cancer.

The zeolitic imidazolate metal organic framework (ZIF-8) has emerged as the most effective and prospective carriers for drug delivery for oncology therapeutics because of its chemical stability under neutral conditions and pH-sensitive structural breakdown under the acidic tumor microenvironment (TME) [14,15]. In addition, ZIF-8 has shown significant advantages over other drug delivery platforms for the following reasons. (1) The large specific surface area and porosity provide the foundation for efficient drug loading; (2) ZIF-8 is easily modified, so various groups can be added for functionalization; (3) ZIF-8 is degradable, so ZIF-8-based nanodrugs are safe with low toxicity and good biodegradability [4,16]. Polyoxometalates (POMs) are important metal–oxygen cluster compounds. They are composed of transition metals such as W and Mo combined with O to form polyanions, and then combined with counterions such as K^+^ and NH_4_^+^ [17]. POMs have been widely studied in the fields of antitumor, antiviral, and antibacterial chemistry with the advantages of strong structural modifiability, low toxicity, few side effects, mature synthetic methods, and low cost. However, there are also some limitations of POMs, such as limited stimulus factors besides pH and GSH dose and a narrow pH range to induce POM quick response under TME and low targeting ability [18,19,20]. Molybdenum (Mo)-based POM clusters exhibit excellent photothermal conversion efficiency under near-infrared laser (NIR) [21]. Meanwhile, POMs with good biocompatibility are expected to show significant ROS scavenging activity, reducing PTT-induced inflammation by transforming reductive Mo^5+^ in POMs into oxidized Mo^6+^ [22,23]. Autophagy enables cancer cells to survive in extreme environments owing to its nutrient and energy supply function [24,25]. In addition, chloroquine, a commonly used antimalarial drug, is a typical autophagy inhibitor that can block the recycling of nutrients and inhibit cancer cell proliferation. Chloroquine diphosphate (CQ), the product of chloroquine modification, shows better hydrophilia than chloroquine and can be easily loaded into hydrophilic drug carriers and used for inhibiting autophagy [26,27].

**Figure 1:**
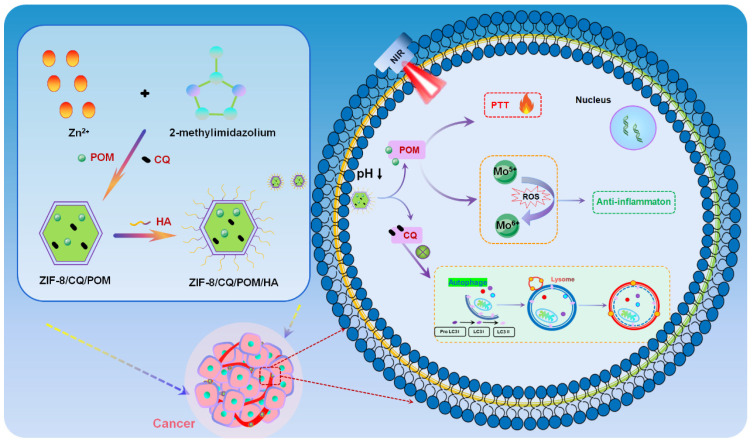
Schematic illustration of the synthesis of ZCPHs and the combined PTT/anti-autophagy/anti-inflammation of tumors.

Herein, we developed a ZIF-8/CQ/POM nanoplatform (defined as ZCP) via one-step synthesis. CQ and POMs are directly encapsulated into ZIF-8 during the synthesis process. Subsequently, hyaluronic acid (HA) with good tumor targeting and biocompatibility is used to coat the surface of the ZCP via electrostatic interaction to form the final nanoplatforms (ZCPHs) [28]. Once in the acidic TME, ZCPHs can release the loaded POMs and CQ. POMs cannot only display considerable photothermal therapeutic capacity under 808 nm near-infrared laser, but also have an impressive anti-inflammatory capacity by scavenging ROS in the TME. The released CQ can be used for lysosome deacidification and autophagy inhibition by cutting off the autophagy-related self-protection pathway caused by cell necrosis. Therefore, this work offers a prospective view on PTT/anti-autophagy/anti-inflammatory combination for efficient ovarian cancer treatment (Figure 1).

## 2. Results and Discussions

### 2.1. Characterization of ZCPHs

**Figure 2:**
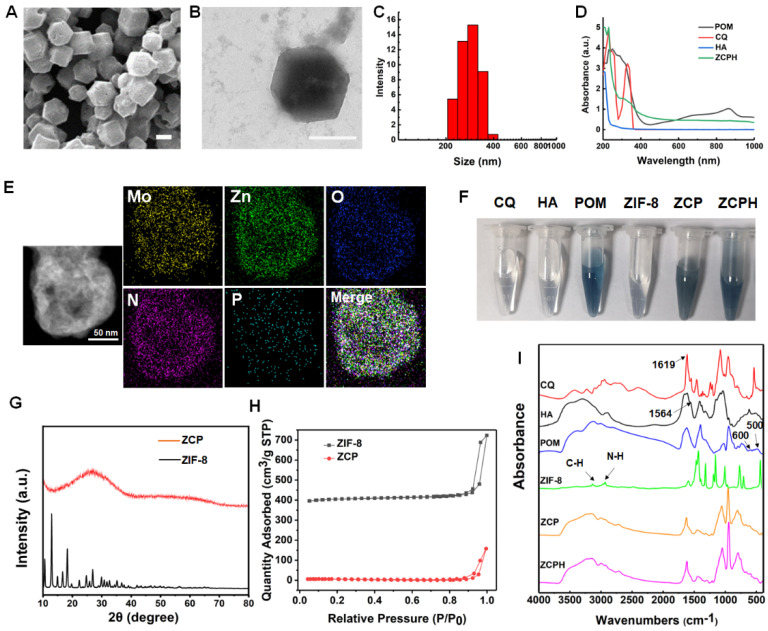
(**A**) SEM images of ZCPHs. (**B**) TEM images of ZCPHs. (**C**) Size distribution of ZCPHs. (**D**) UV-vis-NIR spectra of different groups. (**E**) TEM mapping images of ZCPHs. (**F**) Colors of CQ, HA, POM, ZIF-8, ZCP and ZCPH. (**G**) XRD patterns of the simulated ZIF-8 and ZCP. (**H**) N_2_ absorption-desorption isotherms of ZIF-8 and ZCP. (**I**) FT-IR spectra of CQ, HA, POM, ZIF-8, ZCP and ZCPH. Scale bar: 200 nm.

As shown by Figure 2A–C, ZCPHs showed a regular hexahedral structure with good dispersity, and the DLS results indicated that the average size distribution was about 285 nm. The zeta potential of ZCPHs was determined to be −6.85 mV. The UV-vis spectra of ZCPHs showed that the characteristic peaks of CQ and POMs were 320 and 860 nm, respectively (Figure 2D), indicating that CQ and POMs were successfully encapsulated into ZCPHs. The elemental mapping of ZCPHs showed the uniform distribution of N, O, P, Zn and Mo elements (Figure 2E), indicating that ZCPHs were successful synthesized. During the synthesis process, the color of our nanomaterials turned blue after adding POMs (Figure 2F). From the observation of the XRD patterns of different samples, the intensity of the characteristic peaks of ZCP reduced significantly, which may attribute to the encapsulation of POM with amorphous structure and CQ [29]. The Brunauer–Emmett–Teller (BET) surface areas of ZIF-8 and ZCP were then determined to be 966.7703 and 5.9571 m^2^/g, respectively, demonstrating that abundant CQ and POMs had been successfully loaded into ZIF-8 NPs, reducing the BET surface areas (Figure 2H). The modification processes of nanoplatforms were further qualitatively ascertained by Fourier transform infrared (FT-IR) spectroscopy (Figure 2I). Compared with ZIF-8, CQ, and POMs, ZCP retained all the above characteristic peaks. The original peaks of ZCP at 1619 cm^−1^ can be assigned to the vibrations of aromatic rings in benzene, suggesting the successful incorporation of CQ into ZIF-8. Meanwhile, the vibrational peaks at 500 and 600 cm^−1^ were supposed to be associated with POMs. When compared with HA and ZCP, the peak at 1564 cm^−1^ of ZCPH became wider, which might owe to the existence of the amide bond of HA. The above representative results demonstrated the successful incorporation of CQ and POMs into ZIF-8, successfully modified by HA on the surface.

### 2.2. Photothermal Performance of ZCPHs

**Figure 3:**
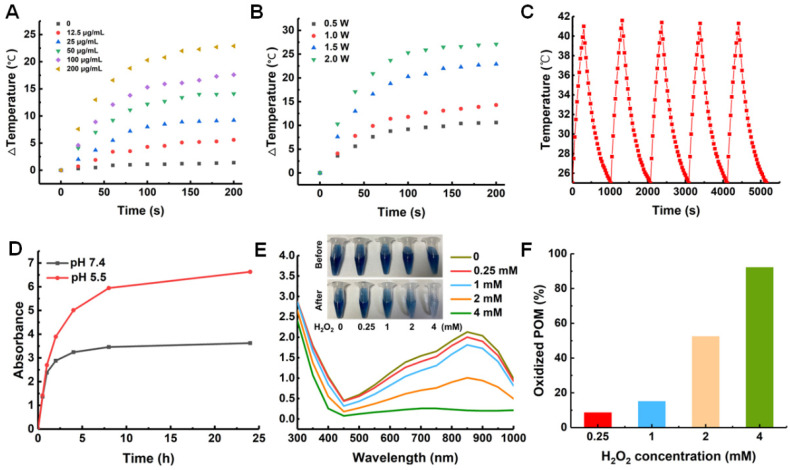
(**A**) Photothermal curves of ZCPH solutions of different doses upon an 808 nm irradiation. (**B**) Photothermal curves of ZCPH solutions upon 808 nm irradiation with different irradiation intensity. (**C**) The heating and cooling curves of ZCPH dispersions over five irradiation cycles. (**D**) Acid-responsive release behavior of CQ after ZCPHs were dispersed in PBS solutions of different pH values (pH = 7.4 and 5.5) at 37 °C. CQ at pH 5.5 condition displayed higher UV absorption than that in pH 7.4 condition. (**E**) The corresponding UV absorbance of POMs before and after incubation with H_2_O_2_ at different concentrations. Insert is the color changes of POMs. (**F**) Quantitative analysis of oxidized POM according to the UV absorption in Figure **E**. The different colors in (**F**) corresponded to that in Figure **E**.

With the prior results we found that the constructed ZCPHs exhibited excellent absorption in the NIR region, which encouraged us to further explore their photothermal conversion behavior. As shown in Figure 3, the irradiation of ZCPHs with 808 nm NIR laser showed an inherent modifiable property of ZCPHs on tumor PTT with increasing ZCPHs dose and NIR optical power density. According to the calculation of photothermal conversion efficiency, the 808 nm laser heat conversion efficiency (ηT) of the ZCPH was 30.8%. Furthermore, no significant changes in the ZCPHs solution temperature were observed after five laser on/off cycles of irradiation (Figure 3C), indicating that ZCPHs have considerable optical and thermal stability.

### 2.3. pH-Responsive CQ Release In Vitro

The drug loading capacity of ZCPHs was measured by UV-vis spectrophotometry after complete disintegration with hydrochloric acid solution. The drug loading of CQ was determined to be about 1%. To confirm the pH-responsive release of CQ, in vitro release was performed in PBS at pH 5.5 and 7.4, respectively. pH 7.4 PBS was used to mimic the normal tissue microenvironment at neutral pH, while pH 5.5 PBS was used to represent the acidic TME. ZCPHs exhibited much faster CQ release in the acidic condition than that of the neutral condition by measuring the absorbance at 320 nm (Figure 3D). The release result that was responsive to pH might be ascribed to the dissolution of ZIF-8 due to low pH [15].

### 2.4. Scavenging ROS with ZCPHs

It is well-established that tumor cells can undergo necrosis when the temperature rises to around 50 °C [30]. PTT has become a promising antitumor approach in the last few years. Although it is quick and effective, PTT is still hobbled by the release of some unwanted inflammatory factors which appear within the TME. These factors could not only affect the treatment effect of immunotherapy, but also hamper PTT by causing inflammation. Therefore, reducing inflammation is one possible way to enhance the therapeutic effects of PTT. It is understood that ROS play a important role in stimulating inflammatory reactions. In this study, the ROS scavenging capability of ZCPHs was further evaluated with H_2_O_2_ as ROS. With the increasing dose of H_2_O_2_ from 0.25 mM to 4 mM, the blue color of the POMs became weak and the intensity of the UV absorption was reduced (Figure 3E). This can be attributed to the fact that Mo^5+^ can be easily oxidized to Mo^6+^ by H_2_O_2_ [23]. The quantitative analysis of oxidized POMs according to the UV absorption indicated that oxidized POMs gradually increased with the increasing H_2_O_2_ doses (Figure 3F). In addition, even the scavenging of 0.25 mM H_2_O_2_ could be easily detected through the decreased UV absorption, indicating that POMs were easily oxidized by H_2_O_2_ and ZCPHs had strong H_2_O_2_ scavenging ability.

### 2.5. In Vitro Combined Therapy

**Figure 4:**
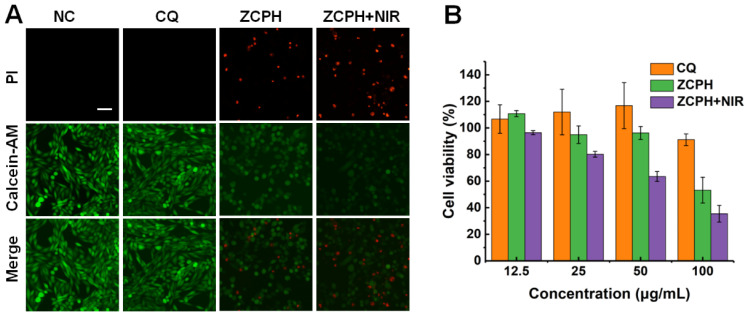
(**A**) The live/dead staining of SKOV3 cells with different treatments. The living cells and dead cells were stained with Calcein-AM and PI, respectively, representing green and red fluorescence. (**B**) SKOV3 cell viability after different treatment. Scale bar: 50 μm.

To demonstrate the cytotoxicity of ZCPHs, MTT assay was performed on SKOV3 cells (Figure 4B). CQ showed minimal toxicity to SKOV3 cells, with over 80% of the cells surviving even at a concentration of up to 100 µg/mL. The ZCPHs enhanced the cytotoxicity of CQ which may be assigned to the high cell uptake. In addition, NIR light significantly enhanced the cytotoxicity of ZCPHs due to their efficient photothermal performance. Subsequently, the local photothermal ablation of SKOV3 cells with ZCPHs under NIR light irradiation was assessed by living/dead cell staining assay (Figure 4A). No obvious dead cells were observed in single NIR light irradiation and CQ. However, SKOV3 cells with ZCPHs treatment displayed obvious cell necrosis, and NIR light further enhanced this effect. Living/dead cell staining assay displayed the same results as that of MTT.

**Figure 5:**
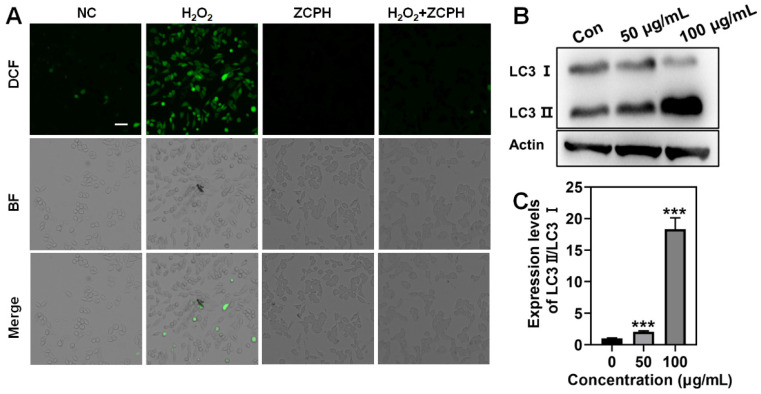
(**A**) Representative ROS staining of SKOV3 cells with DCFH-DA probe. (**B**) Western blotting analysis of LC3 lipidation and (**C**) the corresponding quantitative data after treatment with ZCPHs for 24 h. Scale bar: 50 μm. Statistical significance was calculated via Student’s *t* test, *** *p* < 0.001.

Furthermore, we investigated the ROS scavenging activity of ZCPHs at the cellular level using H_2_O_2_ to establish inflammatory conditions in SKOV3 cells. The build-up of ROS is not only detrimental to healthy normal cells, but also affects the therapeutic outcome of cancer therapy. We found that under the action of H_2_O_2_, cells produced a large amount of ROS, showing strong green fluorescence. After adding ZCPHs on this basis, it was found that the strong green fluorescence in SKOV3 cells weakened because the Mo^5+^ of the POMs of ZCPHs consumed excess H_2_O_2_ (Figure 5A).

### 2.6. Autophagy Efficiency In Vitro

LC3 is the major structural protein of autophagosome and autolysosome, and is extensively supposed to be a marker for detecting autophagy. In the process of completing autophagy, the expression of LC3-I is up-regulated and combined with phosphatidylethanolamine in the cytoplasm to form LC3-II, which is mostly spread in the monomembrane vesicles of autophagosomes. Thus, when lysosomes are dysfunctional, the number of LC3-II will increase and accumulate in autologous lysosomes [31]. The effect of ZCPHs on the autophagy of SKOV3 cells after 24 h treatment was studied by Western blotting analysis. With the increase dose of ZCPHs, the conversion of endogenous LC3-I to the fatty form LC3-II (LC3b phosphatidylethanolamine) increased (Figure 5B,C), revealing the increase of autophagosomes in cancer cells and the occurrence of the anti-autophagy of CQ [32].

## 3. Materials and Methods

### 3.1. Materials

Ammonium molybdate tetrahydrate [(NH_4_)_6_Mo_7_O_24_·4H_2_O], ascorbic acid, and 2-methylimidazolium were purchased from Aladdin Reagents Co., Ltd. (Shanghai, China). CQ was purchased from Beijing Solarbio Science & Technology Co., Ltd. Zinc nitrate hexahydrate [Zn(NO_3_)_2_·6H_2_O], dimethyl sulfoxide (DMSO), anhydrous ethanol, and methanol were purchased from Sinopharm Chemical Reagents Company (Shanghai, China). Hyaluronic acid (HA) was purchased from Macklin Biochemical Technology Co., Ltd. (Shanghai, China). Penicillin–streptomycin, Trypsin–EDTA solution (0.25%), 2, 7-dichlorofluorescein diacetate (DCFH-DA, ≥97%), live/dead cell double staining kit calcein acetoxymethyl ester (calcein AM), and propidium iodide (PI) were bought from Beyotime Biotechnology Co., Ltd. (Shanghai, China). 3-(4, 5-Dimethylthiazol-2-yl)-2 and 5-diphenyltetrazolium bromide (MTT) were purchased from Sangon Biotech (Shanghai, China).

### 3.2. Preparation of POM Clusters

The Keggin-type POM clusters were synthesized by a simple reported method [22]. First, Keggin-type POM clusters were synthesized by gradually dissolving (NH_4_)_6_Mo_7_O_24_ 4H_2_O (5 mmol) in 10 mL of ultrapure water with constant stirring. Then, 5 mL NaH_2_PO_4_·12H_2_O solution (2.92 mmol) was rapidly added to the system. Subsequently, 2 mL ascorbic acid solution (1500 mg/mL) was added dropwise to the system with stirring at 25 °C for 15 min, Finally, 80 mL ethanol was added to precipitate the POM agglomerates and the final product was gained by centrifugation, followed by three washes with water/ethanol and drying in a freeze drier.

### 3.3. Preparation of ZCP NPs

A stock solution of CQ (10 mg/mL) was prepared in deionized water. Zn(NO_3_)_2_·6H_2_O (0.2 g) was dissolved in 0.8 mL of methanol and 2-methylimidazolium (4 g) was dissolved in 8 mL methanol. Subsequently, 4 mL prepared CQ stock solution and 0.033 g POM were incorporated simultaneously into the Zn(NO_3_)_2_ solution under stirring. Then, 8 mL 2-methylimidazolium solution was applied dropwise to the above solution using a burette, and the whole system was stirred continuously for 15 min. The ZCP NPs were finally centrifugated in 9000 rpm, washed as described above, and dried under vacuum at 60 °C in order to obtain the final product.

### 3.4. Preparation of ZCPH NPs

HA (5 mg) was dissolved to transparency in 400 µL deionized water, and then the clear mixture was directly added to 2 mL ZCP NPs methanol solution. After further stirring at 25 °C for 6 h, ZCPHs were precipitated and washed with water by centrifugation (10,000 rpm, 5 min).

### 3.5. Characterization

TEM images were taken using a 120 KV transmission electron microscope (HT7800). UV-visible spectra were recorded with a Genesys 50 spectrometer. Zeta potential and hydrodynamic size were collected on a Malvern Nanosizer. The NIR fluorescence spectroscopy measurement was carried out on a Laser Diode Controller (ADR-1860). XRD were recorded on Rigaku Miniflex 600. FTIR spectra (Nicolet iS5, Thermo Scientific, Waltham, MA, USA) was used to determine the presence of CQ, POM, and HA within the ZIF-8 matrix. Adsorption porosimeter (ASAP 2460, Micromeritics instrument Ltd., Norcross, GA, USA) was applied to measure the surface area of the NPs.

### 3.6. In Vitro PTT

To evaluate the self-adaptive photothermal conversion, ZCPH solutions at six concentration groups (0, 12.5, 25, 50,100, 200 μg/mL) were irradiated with a 808 nm laser at 1.5 W/cm^2^. In a separate study, to investigate the irradiation-dose-dependent photothermal effect, ZCPH solutions at concentrations of 200 μg/mL were irradiated with an 808 nm laser at different irradiation fluence (0.5, 1.0, 1.5,2.0 W/cm^2^). A infrared camera (Cx-Series, FLIR, OR, USA) was employed to record thermal images and measure the temperature of the solutions during the irradiation.

### 3.7. Acid-Responsive Release of CQ

To investigate the acid-responsive release behavior of the nanoparticles, ZCPHs were dispersed in phosphate-buffered saline (PBS) solution at different pH values (pH = 7.4 and 5.5). Then, the solution was centrifuged and the supernatant was retained at predetermined time points. Next, the absorbance of collected aqueous solution at 320 nm was measured utilizing a UV-vis spectrometer (Genesys 50, Thermo Scientific, Waltham, MA, USA).

### 3.8. Cell Culture

Human ovarian cancer SKOV3 cells were cultured in folic-acid-free Dulbecco’s Modified Eagle’s Medium (DMEM), supplemented with 10% fetal bovine serum (FBS) at 37 °C in a humidified atmosphere containing 5% CO_2_, respectively.

### 3.9. In Vitro Cytotoxicity Study

The in vitro cytotoxicity to SKOV3 cells was determined by the MTT method. Briefly, cells were housed in 96-well plates. After adherence overnight, cells were incubated for 24 h in fresh medium containing different concentrations of samples (CQ, ZCPHs, ZCPHs + NIR) with equivalent CQ. MTT solution was added to 96-well plates and after an additional 4 h, all medium was discarded. Finally, DMSO (150 µL) was applied to each well to solubilize the purple insoluble MTT product under shaking. The viability of the cells was tested by a microplate reader at 490 nm.

### 3.10. Live/Dead Cell Staining

SKOV3 cells were placed in 24-well plates and incubated at 37 °C for one full day in a humidified atmosphere containing 5% CO_2_. Then, the cells were treated with CQ, ZCPHs, and ZCPHs + NIR, respectively. In the meantime, normal cells were set as the control group. Meanwhile, the double staining of live and dead cells was performed, and after 24 h of incubation, both cells were stained with Calcein AM and PI, respectively. The changes to cell viability were then detected using a fluorescence microscope.

### 3.11. ROS Scavenging with ZCPHs

SKOV3 cells were seeded into 24-well cell culture plates and then incubated for 24 h. The medium solution of ZCPHs was then added to all wells and incubated for 0.5 h. Cells were then treated with H_2_O_2_ (250 μM) and incubated at 37 °C with 5% CO_2_ for 24 h. Wells with only ZCPHs or cells incubated with H_2_O_2_ only were used as controls. Typical ROS were then stained with DCFH-DA to assess the ROS scavenging activity of ZCPHs at the cellular level.

### 3.12. Western Blotting

Total proteins were isolated from cells by protein extraction reagents according to the standard method and quantified by the BCA Protein Assay Kit. Then, the proteins of all groups were subjected to electrophoresis and membrane transfer. Finally, the target protein was labeled with primary antibody (LC3), where the actin antibody was chosen to be the control group. The color of bands was visualized by ECL detection kit.

## 4. Conclusions

In this study, a multifunctional ZCPH was successfully developed for the combined PTT/anti-inflammation/anti-autophagy of ovarian cancer. The ZCPH exhibited precise targeting to specific marker CD44 on the tumor cell and satisfied dose-dependent toxicity toward SKOV3 cells. More importantly, with the successful encapsulation of POMs and CQ into ZCPH, autophagy and inflammation can be effectively inhibited during PTT, further inhibiting ovarian cancer progression. Therefore, the established ZCPH with outstanding PTT/anti-inflammatory/anti-autophagy capacity provides a promising novel approach for efficient ovarian cancer therapy.

## Data Availability

Not applicable.
